# Collision Tumor in the Pituitary, Concurrent Pituitary Adenoma, and Craniopharyngioma

**DOI:** 10.1155/2020/9584090

**Published:** 2020-09-08

**Authors:** Zaid Shareef, Connor Kerndt, Trevor Nessel, Devin Mistry, Bryan Figueroa

**Affiliations:** ^1^Michigan State University College of Osteopathic Medicine, East Lansing, MI, USA; ^2^Spectrum Health, Michigan State University College of Human Medicine, Grand Rapids, MI, USA; ^3^Department of Otolaryngology, Metro Health Hospital-University of Michigan, Wyoming, MI, USA; ^4^Department of Neurosurgery, Metro Health Hospital-University of Michigan, Wyoming, MI, USA

## Abstract

Collision tumors are two independent, distinct tumors occupying the same anatomical space. This case presents a pituitary adenoma-craniopharyngioma collision tumor presenting with hemianopsia. A 60-year-old with a past history of a nonsecretory pituitary adenoma presented with progressive headaches, bitemporal hemianopsia, and nausea. Previously, in 2008, his adenoma was effectively treated with nasal septal flap and transsphenoidal pituitary resection. A magnetic resonance imaging (MRI) was ordered for concern of recurrence, given his history and neurologic complaints. The MRI revealed a suprasellar mass extending into the third ventricle with displacement of the hypothalamus and optic chiasm. Laboratory testing revealed no indicators of endocrinopathy. The neurosurgical and otolaryngologic teams were elected to perform tumor resection given the ongoing symptoms. An image-guided transsphenoidal tumor resection with abdominal fat graft harvest and septal mucosal flap CSF leak repair was performed. Histopathological examination revealed two tumor components within the resection including an adamantinomatous craniopharyngioma and recurrent pituitary adenoma.

## 1. Introduction

Collision tumors are defined as two independent, histologically distinct tumors that occupy the same anatomical space. Suprasellar collision tumors, specifically pituitary gland tumors within the sella turcica, display a remarkable variety of malignancy. The coexistence of a pituitary adenoma with craniopharyngioma is an exceptional clinical finding. A pituitary adenoma is the most common intracranial sellar mass, comprising 10–15% of all intracranial tumors, while craniopharyngiomas account for 1.0–4.0% of intracranial tumors [1]. Craniopharyngiomas are exceptionally rare, occurring at a rate of 1.3 tumors per million persons per year. Recent literature review revealed only 15 other cases of pituitary adenoma-craniopharyngioma collision tumors to date [2, 3]. This case presents a 60-year-old male who underwent the image-guided endoscopic transsphenoidal approach to the middle cranial fossa skull base with a complex abdominal fat graft harvest and septal mucosal flap with cerebral spinal fluid (CSF) leak repair.

## 2. Case Presentation

A 60-year-old left-handed male with a past medical history of a pituitary adenoma (2.5 cm × 2.0 cm), type-2 diabetes mellitus, hypertension, and hyperlipidemia presented to his optometrist with complaint of nonremitting visual deficit. On further examination, the optometrist diagnosed the patient with a bitemporal visual field deficit. Due to the patient's previous history, the optometrist recommended follow-up with his primary care provider (PCP) for further investigation.

A week later, he presented to his PCP with the continued complaint of progressively worsening headaches and visual changes outlined above. The patient reported a four-week-history of daily, visual disturbance with associated headache, imbalance, and nonintractable nausea. Of note, his past surgical history was significant for nasal septal flap and transsphenoidal pituitary resection in 2008 for primary pituitary adenoma. He failed to receive postoperative imaging surveillance after this procedure as he was lost to follow-up.

**Figure 1:**
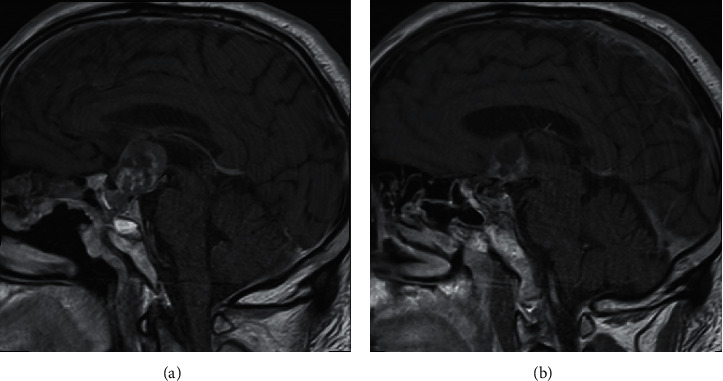
Initial MRI without gadolinium ordered by primary care provider, revealing a suprasellar mass extending into the anterior portion of the third ventricle and displacing the hypothalamus. There is evidence of a robust septal flap and residual fat graft from prior resection.

Neurological examination revealed slight bitemporal visual field deficit on confrontation and was otherwise unremarkable. On examination, his vital signs revealed a temperature of 98.2 F, blood pressure of 120/74 mm of Hg, and heart rate of 84 bpm, and SpO_2_ was 93%. Due to previous history, lack of surveillance, and progressive symptoms, brain imaging was obtained. An MRI was ordered by the primary care provider. An MRI without gadolinium revealed a heterogeneous 3 cm suprasellar mass extending into the third ventricle, with superior displacement of the hypothalamus and optic chiasm (Figure 1).

**Figure 2:**
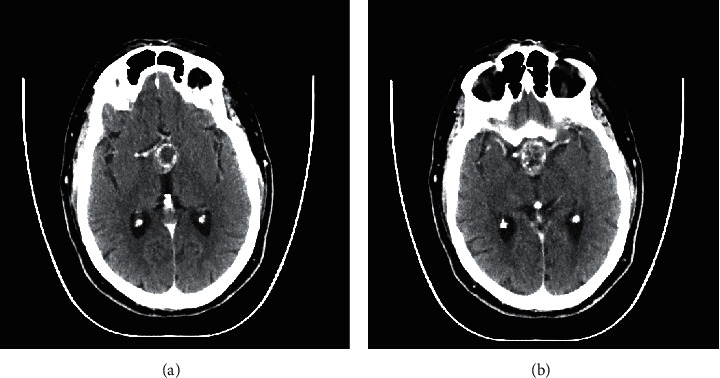
CT with contrast obtained by neurosurgery and otolaryngologist the day before surgery revealing a 2.7 cm × 2.3 cm × 1.9 cm suprasellar mass with peripheral calcifications. The mass impinges on the foramen of Monro and the anterior portion of the third ventricles.

Subsequently, after undergoing intracranial imaging by his PCP, he was referred to neurosurgery and otolaryngology for recurrence of pituitary adenoma and consideration of endoscopic skull base surgery. Further laboratory testing demonstrated no evidence of endocrinopathy with hormone levels within normal limits. These included assessing levels of prolactin, insulin-like growth factor 1 (IGF-1), luteinizing hormone (LH), follicle-stimulating hormone (FSH), thyroid release hormone (TRH), cortisol, and T4. Thereafter, the patient received a CT scan prior to surgery revealing the tumor invading into the third ventricle (Figure 2).

**Figure 3:**
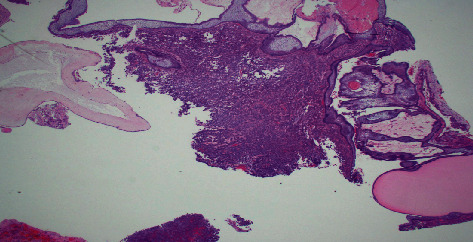
Histological examination revealing collision tumor transition point. The center of the image reveals pituitary adenoma tissue with elongated cells in a pseudorosette pattern. The surrounding tissue reveals the adamantinomatous craniopharyngioma with whorls of keratin and stellate reticulum.

**Figure 4:**
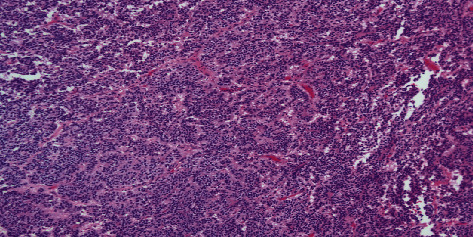
This histological section reveals a higher powered magnification of Figure 3 demonstrating the pituitary adenoma with elongated cells in a pseudorosette pattern.

**Figure 5:**
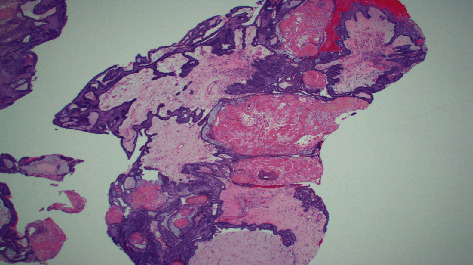
This section reveals stellate reticulum in the middle of the image with whorls of keratin diffusely throughout consistent with the adamantinomatous craniopharyngioma.

Image-guided endonasal transsphenoidal anterior skull base tumor resection with abdominal fat graft harvest and septal mucosal flap CSF leak repair was performed. Subsequent histopathological examination of the surgical specimen revealed two tumor components within the resection: adamantinomatous craniopharyngioma and hypercellular recurrent pituitary adenoma (Figure 3). The pituitary adenoma portion revealed elongated cells in a pseudorosette pattern which was expected for this type of tumor (Figure 4). The craniopharyngioma component showed diffuse strong nuclear staining with p40 and cytoplasmic staining with CK7. Histology of the adamantinomatous craniopharyngioma revealed stellate reticulum with whorls of keratin (Figure 5). Synaptophysin and chromogranin strongly stained the pituitary cells. Reticulin stain showed a disrupted reticulin network in the area of monotonous pituitary cells.

Postoperatively, the patient developed diabetes insipidus overnight, which required two doses of oral administration (PO) of desmopressin (DDAVP). The only visual complaint was diplopia. Otherwise, there were no other abnormalities. The next day, the patient was cleared to work with physical therapy and occupational therapy. That same day, he again met criteria for DI and received a third dose of DDAVP. Due to this, the patient was started on 2 micrograms (mcg) DDAVP PO BID, and his follow-up sodium was 135 mEq/L. After this, his sodium level warranted to decrease his DDAVP dose to 1 mcg PO BID. On the patient's fourth postoperative day, his sodium levels were in the normal range. He was discharged with a repeat sodium the following week which showed to be in normal ranges. No other endocrinological abnormalities were noted postoperatively. The patient was scheduled for serial follow-ups in the next upcoming months and was informed about the possible symptoms caused by developing sodium imbalances.

## 3. Discussion

Collision tumors are a rare subset of tumors, especially when located in the sella turcica. To date, literature review yields only fifteen previously documented cases of collision tumors of pituitary adenoma with coexisting craniopharyngioma. Both pituitary adenomas and craniopharyngiomas are benign tumors that arise from the adenohypophysis. Pituitary adenomas are the most common causes of sellar masses, which typically arise in the third decade of life [3]. Craniopharyngiomas are hypothesized to develop from remnants of Rathke's pouch and display bimodal age distribution. In children, they most commonly arise between the ages of 5 and 14 and in adults most commonly between ages 50 and 75.

There are two known theories regarding the presence of craniopharyngiomas. Embryonal craniopharyngiomas are generally consistent with the adamantinomatous (AD) subtype of craniopharyngiomas, which are more commonly seen in the pediatric population. The other subtype is metaplastic papillary craniopharyngiomas, which are primarily seen in the adult population. This case differs from the current paradigm as this patient's histology revealed an adamantinomatous subtype of craniopharyngioma instead of a metaplastic papillary craniopharyngioma, which would be expected based on this patient's age [4].

The embryonal model describes the more common subset, which is found predominantly in infants and young adolescents. This paradigm conjects that this tumor originates from defects in embryogenesis, where remnants of Rathke's pouch fail to involute, and residual tissue remains in the sella turcica, resulting in the formation of a craniopharyngioma [5]. Given its embryologic origin, the AD subtype preferentially affects the pediatric population.

In contrast, the metaplastic model of craniopharyngioma origin is suggested to be a result of a metaplastic process of the anterior pituitary. Specifically, the cells of the pars tuberalis undergo a metaplastic process that allows squamous cells to proliferate and develop into a papillary craniopharyngioma [5]. This papillary subtype of craniopharyngioma inflicts older, adult populations as the metaplastic processes take time to occur, which differs from the AD subtype.

Diagnosis and treatment of pituitary collision tumors are frequently challenging because of the limited space for operation and biopsy. Additionally, the predilection of these tumors to adhere to surrounding structures and ability to recur make surgical intervention complicated. Given the small potential space of the sella, the tumor frequently extends into the pituitary stalk, hypothalamus, third ventricle, cerebellopontine angle, and subtemporal area [6]. In this patient's case, the tumor outgrew its potential space and encroached into the third ventricle as a result. Due to the nature of ventricular invasion of the tumor in this patient, resection had a high likelihood of causing a CSF leak, thus further complicating surgical resection and necessitating CSF leak repair.

Some debate whether surgical resection of a recurring pituitary tumor via the transsphenoidal approach is appropriate. Literature suggests that craniotomy is the superior approach for large complex pituitary tumors versus endoscopic transsphenoidal resection in reducing the risk of tumor recurrence [7]. Ideology behind this suggests that exploration of the suprasellar region allows for enhanced visualization of the coexistence of further tumor burden. However, the transsphenoidal approach has gained recent favor as a result of its reduced intraoperative and postoperative complication rates. Endoscopic transsphenoidal surgery has an associated 22% reduction in developing postoperative diabetes insipidus, which is a common postoperative complication [6]. The techniques necessary for transsphenoidal intervention requires extensive otolaryngologic and neurosurgical training to successfully perform; however, data suggest that this results in improved surgical patient outcomes [8]. Endoscopic transsphenoidal surgery is associated with higher gross tumor removal and a lower incidence of septal perforation [7].

Due to the limited literature on endoscopic skull base approaches, it is also questioned whether endoscopic transsphenoidal approaches are superior to microscopic approaches. Both produce relatively similar outcomes and complications, yet the endoscopic procedure does allow better visualization of the tumor and surrounding structures [9]. It also has been shown that the endoscopic approach shows superb visual recovery, which was this patient's initial primary complaint. The selection of surgical approach primarily depends on the skillset of the physician and their previous experience performing the specific surgical approach [9].

Despite better outcomes with transsphenoidal surgery, many cases of pituitary tumors have displayed recurrence of their primary tumor. This patient initially presented with a diagnosis of pituitary adenoma and progressed to have recurrence of this tumor compounded with the addition of a craniopharyngioma, leading to the diagnosis of pituitary adenoma-craniopharyngioma.

Interestingly, other cases have also described tumor recurrence as a pathway to collision tumor [10]. This brings to question the initial findings upon primary resection. It is possible that the original tumor was a true collision tumor that was not identified given the histopathologic sample. The diagnosis of the adamantinomatous subtype, as in this case, is generally aligned with a model of embryological development. However, the craniopharyngioma was not identified on the first resection, which confounds the origin of this tumor. It further questions whether primary resection failed to reveal its distinct histopathology or whether this form of the adamantinomatous subtype was a result of the metaplastic model or an unknown development.

## 4. Conclusion

The presence of a collision tumor in the pituitary region including a pituitary adenoma and craniopharyngioma is an exceptional clinical finding. Diagnosis is difficult, as CT scan and MRI have difficulty in distinguishing between two distinct tumors and other mimicking pathologies. Definitive diagnosis requires histopathological examination demonstrating the presence of 2 distinctly different tissues. Early identification with clinical suspicion and diagnosis can prevent invasive treatment via craniotomy and allow for more efficacious endonasal transsphenoidal approach for tumor resection.

## Data Availability

The data used to support the findings of the study are available from the corresponding author.

## References

[1] Gsponer J., Tribolet N. D., Déruaz J.-P. (1999). Diagnosis, treatment, and outcome of pituitary tumors and other abnormal intrasellar masses: retrospective analysis of 353 patients. *Medicine*.

[2] Reymond T., Kowari K., Eda H., Kambara M., Maruyama R., Akiyama Y. (2019). Ten-year follow-up of collision tumors composed of craniopharyngioma and pituitary adenoma: a case report and literature review. *Case Reports in Medicine*.

[3] Alexander J. M., Biller B. M., Bikkal H., Zervas N. T., Arnold A., Klibanski A. (1990). Clinically nonfunctioning pituitary tumors are monoclonal in origin. *Journal of Clinical Investigation*.

[4] Louis D. N., Perry A., Reifenberger G. (2016). The 2016 world health organization classification of tumors of the central nervous system: a summary. *Acta Neuropathologica*.

[5] Ohgaki Y., Ni M., Wang Y., Zhong L. (2018). Comparison of neuroendocrine dysfunction in patients with adamantinomatous and papillary craniopharyngiomas. *Experimental and Therapeutic Medicine*.

[6] Qi W., Gu F., Wu C. (2019). Growth hormone replacement therapy improves hypopituitarism-associated hypoxemia in a patient after craniopharyngioma surgery: a case report. *Medicine*.

[7] Li A., Liu W., Cao P., Zheng Y., Bu Z., Zhou T. (2017). Endoscopic versus microscopic transsphenoidal surgery in the treatment of pituitary adenoma: a systematic review and meta-analysis. *World Neurosurgery*.

[8] Prete A., Corsello S. M., Salvatori R. (2017). Current best practice in the management of patients after pituitary surgery. *Therapeutic Advances in Endocrinology and Metabolism*.

[9] Wang E. W., Gardner P. A., Zanation A. M. (2019). International consensus statement on endoscopic skull-base surgery: executive summary. *International Forum of Allergy & Rhinology*.

[10] Jin G., Hao S., Xie J., Mi R., Liu F. (2013). Collision tumors of the sella: coexistence of pituitary adenoma and craniopharyngioma in the sellar region. *World Journal of Surgical Oncology*.

